# Vector maps and spatial autocorrelation of carbon emissions at land patch level based on multi-source data

**DOI:** 10.3389/fpubh.2022.1006337

**Published:** 2022-10-20

**Authors:** Xiaoping Zhang, Qinghua Liao, Hu Zhao, Peng Li

**Affiliations:** ^1^School of Architecture and Urban Planning, Shandong Jianzhu University, Jinan, China; ^2^School of Architectural Engineering, Tongling University, Tongling, China; ^3^Zibo Urban Planning Design Institute Co., Ltd., Zibo, China

**Keywords:** vector maps, spatial autocorrelation, carbon emissions, multi-source data, land patch, Zhangdian

## Abstract

An accurate carbon emissions map is of great significance for urban planning to reduce carbon emissions, mitigate the heat island effect, and avoid the impact of high temperatures on human health. However, little research has focused on carbon emissions maps at the land patch level, which makes poor integration with small and medium-sized urban planning based on land patches. In this study, a vectorization method for spatial allocation of carbon emissions at the land patch level was proposed. The vector maps and spatial autocorrelation of carbon emissions in Zhangdian City, China were explored using multi-source data. In addition, the differences between different streets were analyzed, and the carbon emissions ratio of the land patch was compared. The results show that the vector carbon emissions map can help identify the key carbon reduction land patches and the impact factors of carbon emissions. The vector maps of Zhangdian City show that in 2021, the total carbon emissions and carbon absorptions were 4.76 × 10^9^kg and 4.28 × 10^6^kg respectively. Among them, industrial land accounted for 70.16% of carbon emissions, mainly concentrated in three industrial towns. Forest land carbon absorption accounted for 98.56%, mainly concentrated in the peripheral streets away from urban areas. The Moran's I of land patch level carbon emissions was 0.138, showing a significant positive spatial correlation. The proportion of land patches is an important factor in determining carbon emissions, and the adjustment of industrial structure is the most critical factor in reducing carbon emissions. The results achieved can better help governments develop different carbon reduction strategies, mitigate the heat island effect, and support low-carbon and health-oriented urban planning.

## Introduction

Urban planning is an important means to control carbon emissions. Urban planning can improve energy efficiency, reduce carbon emissions, mitigate the heat island effect and promote public health through rational allocation of land resources (1, 2). Studies have shown that urban areas account for 76% of primary energy consumption, resulting in carbon emissions and ambient air pollution (3). Air pollution is one of the most important causes of global human health risks (4). In addition, urban planning can reduce energy carbon emissions by 12% by adjusting land use structure (5). Compared to a comfortable temperature of 23°C, each 1°C increase in temperature was associated with a predicted 0.2% increase in psychological stress (6). Therefore, urban planning is a key technology for reducing carbon emissions and promoting public health.

The carbon emissions map is an important basis for urban planning to reduce carbon emissions. Urban planners can intuitively obtain the characteristics of carbon emissions within the research scope (7, 8), identify the hot and cold spots of carbon emissions, and then formulate targeted low-carbon and health-oriented urban planning scheme (9, 10). For example, Guttikunda et al. mapped Madrid's carbon emissions based on a geographic information system, and analyzed the spatial characteristics of carbon emissions, as well as the effects of buildings numbers and population density (11). By mapping carbon emissions, Oliveira found that urban planning elements such as urban functional layout, development intensity, road network density, public green space, and housing environment affect the distribution and diffusion of energy consumption and air pollutants, which in turn affect residents' health (12). Zhang et al. analyzed the spatial distribution of urban carbon emissions by mapping land use carbon emissions and proposed a targeted low-carbon urban planning strategy (8). Since carbon emissions maps can be used as a scientific means to support low-carbon healthy urban planning, there is a growing number of studies focusing on the establishment of carbon emissions maps.

At present, the establishment of carbon emissions maps mainly includes three methods: (1) Based on statistical data, a comparative study of the spatial heterogeneity of different administrative divisions is carried out at the macro level. For example, the European Union's Emissions Database for Global Atmospheric Research (EDGAR) (13), Purdue University's Fossil Fuel Data Assimilation System (FFDAS) (14), China High-Resolution Emission Database (CHRED) (15, 16). The spatial resolution of the above databases is mainly 10km × 10km grid, which is not suitable for spatial analysis of specific objects due to the relatively coarse resolution (14). (2) The greenhouse gas (GHG) inventory is assigned to a regular grid using gridded data that approximate the location and intensity of human activities, such as population (17), nighttime lighting (18), and gross domestic product (GDP) [(19), Yang et al., 2021]. For example, the Carbon Dioxide Information Analysis Center (CDIAC) used population density to classify emissions. The results were shown as a 1° × 1°grid carbon emissions map (20). Zhao et al. simulated the spatial distribution of global carbon emissions with a resolution of 1 × 1 km through night light data (21). Gao et al. integrated the data of population, GDP, and night lighting, and constructed a spatial allocation grid of carbon emissions of 10 × 10 km. The results showed that the carbon emissions map generated by the spatial allocation model constructed with the three kinds of data is closer to the actual situation (22). However, since the resolution of a gridded carbon emissions map is usually determined by the resolution of grid allocation parameters, it lacks accuracy when applied at the urban scale and is poorly combined with urban planning (23, 24). For example, the industry is the main source of carbon emissions, but large enterprises with high energy consumption are generally located on the edge of cities, with low population density and low night lighting intensity (25). Therefore, it is difficult to identify high carbon emissions areas only through the grid data of population and night lighting (26). (3) Spatial allocation of carbon emissions by land use type based on statistical yearbooks or greenhouse gas (GHG) inventories, also known as vector carbon emissions map. Since the vector carbon emissions map can maintain the accuracy of carbon emissions estimation from administrative units to land patches and can be better integrated with urban planning, more and more scholars have explored how to establish vector carbon emissions map. For example, Zhang et al. established the corresponding relationship between GHG inventory and different land use types and drew the vector map of land use carbon emission according to the average carbon emissions intensity of different land use types, which was used to guide the low-carbon planning (8). Chuai et al. allocated carbon emissions from industrial, commercial, and residential sectors to different land use types according to different allocation parameters of big data, and obtained the spatial distribution of land use carbon emissions (27). Liu et al. divided the carbon emissions in greenhouse gas (GHG) inventory into three categories: points, lines, and areas, and created an algorithm to decompose these emissions into basic objects to obtain a vector carbon emissions map (28). These studies laid the foundation for the establishment of a vector carbon emissions map. However, the above-mentioned carbon emissions map drawing method may need to be improved in three main aspects: first, there are more studies on the scale of administrative divisions, but less attention is paid to the basic unit of the land patch, which makes the integration with small and medium-sized urban planning with land patches as the basic unit poor. Second, the vectorization method for spatial allocation of carbon emissions at the land patch level has not yet been established, which makes the results of these studies inaccurate when applied at the land patch level, thus multi-source data can be used to find detailed geographic objects as vector elements for spatial allocation. Third, it is impossible to distinguish the carbon emissions differences of detailed land use types within cities, such as urban residential land, rural residential land, commercial land, business land, etc., there is insufficient research on the spatial autocorrelation of carbon emissions at the land patch level.

In view of the current gap, this study takes Zhangdian City, China as an example, and uses multi-source data to explore the vector map and spatial autocorrelation of carbon emissions at the land patch level. The reliability and accuracy of the research results are proved by comparison with other research methods. Specifically, the main contributions of this study are as follows. First, a vectorized method for spatial allocation of carbon emissions at the land patch level is proposed and a map of carbon emissions at the patch level including detailed land use types is established, which complements the existing research. In addition, this study also considers the spatial autocorrelation of carbon emissions at the land patch level, aiming to make an important contribution to the existing literature. Overall, this study can provide new theoretical and practical guidance for high-resolution carbon emissions simulation, better serve the government to formulate differentiated carbon emission reduction strategies and provide support for low-carbon and health-oriented urban planning. The study begins by emphasizing in the first section the significance of establishing the vector carbon emissions map and the knowledge gaps in the literature. The second section explains the study area, research data, and methods. The third section divided the findings into two parts: vector carbon emissions map based on land patches and spatial autocorrelation of carbon emissions. The fourth section further discusses the applications, limitations, and further improvements of the results. The fifth section presents the conclusions.

## Materials and methods

### Study area

**Figure 1:**
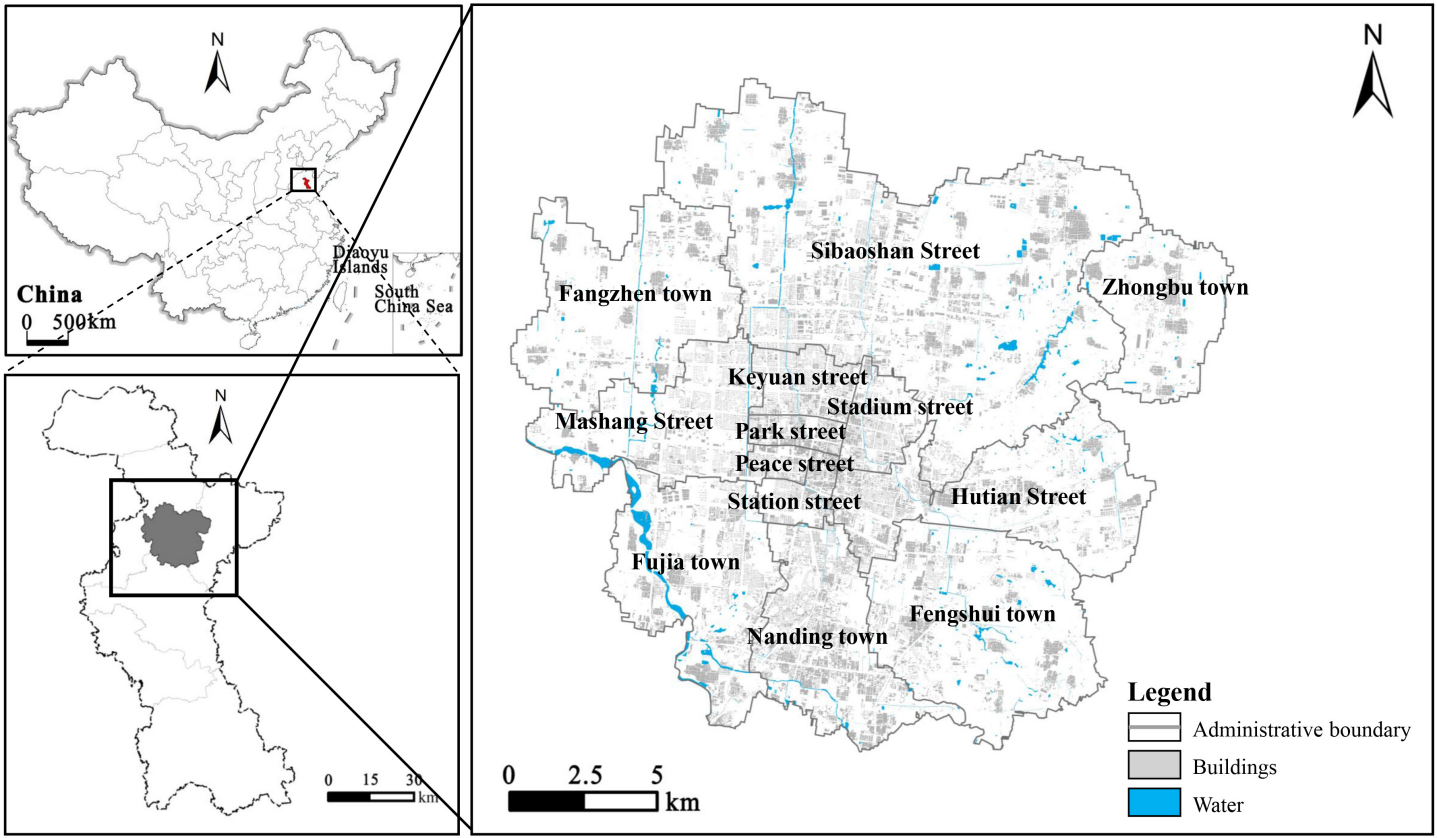
Location of the area of this study.

Zhangdian (35° 55'-37° 17' N, 117°32'-118°31' E) is the political, economic, and cultural center of Zibo city, located in the east of China. At the end of 2021, Zhangdian covers an area of 360 km^2^. With a permanent population of 795,800 and an urbanization rate of 96%. It has jurisdiction over 13 streets, including Hutian Street, Nanding Town, Sibaoshan Street, Fengshui Town, Fujia Town, Fangzhen Town, Stadium Street, Mashang Street, Zhongbu Town, Peace Street, Park Street, Station Street, and Keyuan Street. Among them, Nanding, Sibaoshan, and Fengshui have a relatively solid industrial foundation. Mashang is the core area with the most concentrated population and activities in Zhangdian, and its economic level is relatively high, while other areas have relatively low economic and social capacity. Zhangdian has always been a typical resource-based city with high energy consumption and high carbon emissions, facing urgent demand and huge potential for low-carbon transformation and development. Therefore, choosing Zhangdian as the study area has certain typicality (Figure 1).

### Research data

The multi-source data used include (1) the 2021 Zibo statistical yearbook that provides energy consumption and other economic and social data on Zhangdian. (2) The point of interest (POI) data of all industrial enterprises in Zhangdian were obtained, totaling 2,421 items, including enterprise longitude and latitude information, address name, enterprise name, and enterprise type, which were obtained from the Baidu Map platform with high accuracy and effectiveness. (3) Road system data. The area and length data of different grade roads in Zhangdian were obtained. (4) Land use type data. The land use data of Zhangdian were obtained, including urban residential land, rural residential land, commercial land, industrial land, road, green land, water, arable land, etc., which were provided by Zibo Natural Resources and Planning Bureau. (5) Buildings data. The number of building floors, building area, and building types on all kinds of land was obtained (Table 1).

**Table 1:** Data sources and description.

**Name**	**Description**	**Sources**
Industrial POI data	Industrial storage land patch spatial location dataset	Baidu map
Road systems of Zhangdian	Vector data of road traffic system	Department of transportation
Land use map of Zhangdian in 2021	Vector map of different land use types	Natural resources bureau
Urban population data	Population of urban residential lands	Public security bureau
Rural population data	Population of rural residential lands	Public security bureau
Residential land data	Area, location, perimeter, green, and function	Natural resources bureau
Agricultural planting area	Area, location, type, etc. of agricultural planting	Natural resources bureau
Architecture data	Building outline contains name, number, height, area, perimeter, and floor information	Construction bureau
Electricity consumption data	Electricity consumption of Zhangdian	Statistics bureau

### Methods

The 2021 Zibo statistical yearbook provides energy consumption data. First, energy consumption is allocated to different types of land patches based on different influencing factors (29, 30). Second, carbon emissions are calculated by the carbon emissions factor method (31). Third, Mapping the vector carbon emissions and absorption maps. Finally, spatial patterns are analyzed by spatial autocorrelation.

#### Vectorization methods for spatial allocation of carbon emissions

##### Industrial emissions allocation method

In this study, according to different allocation methods, carbon emissions are mainly divided into three major categories: industrial, road traffic, and other types of carbon emissions (agricultural, residential and commercial carbon emissions, etc.). Carbon absorption mainly refers to ecological carbon absorption. In the 2021 Zibo Statistical Yearbook, enterprises energy consumption is calculated separately according to different industries, which is quite different from other energy consumption (32, 33). In this study, POI information of all enterprises was obtained through the Baidu Map platform. These POI points, with precise latitude and longitude coordinates and enterprise names, can be used as basic objects for space allocation (34). Based on the energy consumption data of different industries and the number of enterprises in different industries provided in the 2021 Zibo statistical yearbook, the weight is calculated and allocated to different enterprises (27, 35). Among them, the energy consumption data of different industries contains the sum of the static energy consumption of enterprises. Some enterprises generate energy consumption in industrial production process, but it is ignored because the number of such enterprises is small. Overall, the energy consumption data of different industries can represent industrial carbon emissions to some extent. The formula of industrial emissions allocation is shown in Equation (1).


(1) $$\begin{array}{l} C_{ij}=C_{ej}\times \frac{E_{ij}}{E_{j}} \end{array}$$

Where *C*_*ij*_ is the carbon emissions of enterprise i of industry j, *C*_*ej*_ is the total carbon emissions of industry j. *E*_*ij*_ is the energy consumption of enterprise i of industry j, *E*_*j*_ is the total energy consumption of industry j.


(2) $$\begin{array}{l} C_{ej}=AD\;\times \;EF \end{array}$$

Where *C*_*ej*_ is carbon emissions (kg), AD is electricity consumption (kW h), EF is carbon emissions coefficient (CO_2_/kW h). On the one hand, in the 2021 Zibo statistical yearbook, energy consumption is uniformly converted into electricity energy consumption (kW H), such as industrial energy consumption, transportation energy consumption, residential energy consumption, etc., which can easily compare the differences in energy consumption of different departments. Therefore, the carbon emission coefficient of energy consumption is consistent. On the other hand, there is no separate carbon emission coefficient of power consumption in Zhangdian. This study obtains the data from the China Energy Statistical Yearbook, which can reflect the local situation to a certain extent. Therefore, AD is the electricity consumption provided by the 2021 Zibo statistical yearbook. EF is quoted from the China Energy Statistical Yearbook, and the value is 1.246 kg CO_2_/kW h.

##### Road traffic emissions allocation method

The road traffic carbon emissions mainly depend on the driving distance and the number of vehicles (36, 37). Among them, the driving distance depends on the length of the road. The number of vehicles depends on the traffic flow, and the traffic flow depends on the road grade (28). This study divides roads into the regional road, the urban road, and the rural road. The traffic flow of the road is calculated according to the average value, as shown in Table 2.

**Table 2:** The reference value of the traffic flow of each grade of the road.

**Road grade**	**Regional road**	**Urban road**	**Rural road**
Traffic flow (Standard vehicles per hour)	4,500	2,067	500

Firstly, according to the traffic flow and road area of different roads, the carbon emissions ratio of different roads is calculated. Secondly, the carbon emissions of each grade road are assigned to each section according to the weight of the road area (38–40). The formulas of road traffic carbon emissions allocation are shown in Equation (3) and Equation (4).


(3) $$\begin{array}{l} C_{ij}=C\times \beta_{j}\times \frac{S_{ij}}{S_{j}} \end{array}$$

Where *C*_*ij*_ is the carbon emissions of segment i of the road grade j, *C* is the total carbon emissions of the road system, *S*_*ij*_ is the area of segment i of the road grade j, *S*_*j*_ is the total area of the road grade j, β_*j*_ is the proportion of carbon emissions in total traffic emissions of grade j.


(4) $$\begin{array}{l} \beta_{j}=\frac{Q_{j}\times S_{j}}{\overset{n}{\underset{j=1}{\sum }}\left(Q_{j}\times S_{j}\right)},\left(n=3\right) \end{array}$$

Where *S*_*j*_ is the total area of the road grade j, *Q*_*j*_ is the traffic flow of the road grade j.

##### Other emissions allocation method

Agriculture, residential and commercial carbon emissions account for a relatively high proportion, and the spatial distribution is relatively concentrated, with good continuity, and the calculation method is relatively consistent. Therefore, agricultural, residential, and commercial carbon emissions were defined as other types of carbon emissions (28). In this study, other types of carbon emissions were allocated to arable land, urban residential land, rural residential land, commercial land, and other land patches by building type, building area, land use area, and other parameters.

Agricultural activities carbon emissions include rice, fertilizer use, intestinal fermentation, and manure management. The agricultural carbon emissions in this study were obtained through the agricultural carbon emissions coefficient and agricultural planting area (41, 42). The formula of agricultural activities carbon emissions allocation is shown in Equation (5).


(5) $$\begin{array}{l} C_{i}=C_{e}\times A_{i} \end{array}$$

Where *C*_*i*_ is the carbon emissions of land patch i of arable land, *C*_*e*_ is the carbon emissions coefficient of arable land, *A*_*i*_ is the area of land patch i of arable land.

Residential and commercial sectors carbon emissions were allocated in similar ways. Taking urban residential land as an example, the energy consumption of urban residents was obtained according to the 2021 Zibo statistical yearbook and then allocated to each residential land based on the buildings area (2, 43, 44). Among them, the energy consumption of urban residential land mainly includes buildings, transportation, waste treatment, etc. Among them, energy consumption accounts for the largest proportion (30). The actual energy consumption includes central heating, electricity, water, and gas, which is mainly used for heating, cooling, lighting, equipment, etc. Electricity consumption and natural gas consumption are the main source of carbon emissions, of which electricity consumption accounts for more than 95 %, while the natural gas consumption is generally within 1 % (45). Many urban lands such as commercial land does not even use natural gas consumption. Other land types, such as rural residential land, commercial land, etc., were calculated using the same method. Therefore, the residential and commercial sectors carbon emissions can be mainly characterized by electricity consumption carbon emissions. The carbon emissions of each land use type were assigned to the corresponding land patches based on the weight of the buildings area or land area. The formula of residential and commercial sectors carbon emissions allocation is shown in Equation (6).


(6) $$\begin{array}{l} C_{i}=C\times \frac{S_{i}}{\sum S_{i}} \end{array}$$

Where *C*_*i*_ is the carbon emissions of land patch i of urban residential land, rural residential land, commercial land, etc. *C* is the total carbon emissions of urban residential land, rural residential land, commercial land, etc. *S*_*i*_ is the area of land patch i.

##### Ecological absorption allocation method

The carbon absorption system includes forest land, grassland, unused land, and water. Different land types have different carbon absorption coefficients (46). Among them, the carbon absorption coefficients are determined according to the IPCC (47) (Table 3), while the area of different land types is determined according to the 2021 Zibo statistical yearbook and Zhangdian territorial space planning.

**Table 3:** Carbon absorption coefficient of land use (tCO_2_/hm^2^.a).

**Type**	**Forest land**	**Grassland**	**Water**	**Unused land**
Coefficient of carbon absorption	0.6125	0.0205	0.0253	0.005

The formula of carbon absorption is shown in Equation (7).


(7) $$\begin{array}{l} C_{i}=C_{ei}\times A_{i} \end{array}$$

Where *C*_*i*_ is the carbon absorption of land patch i, *C*_*ei*_ is the carbon absorption coefficient of land patch i. *A*_*i*_ is the area of land patch i. Among them, *C*_*e*_ is quoted from the IPCC.

#### Mapping land patch level carbon emissions and absorption based on GIS

**Figure 2:**
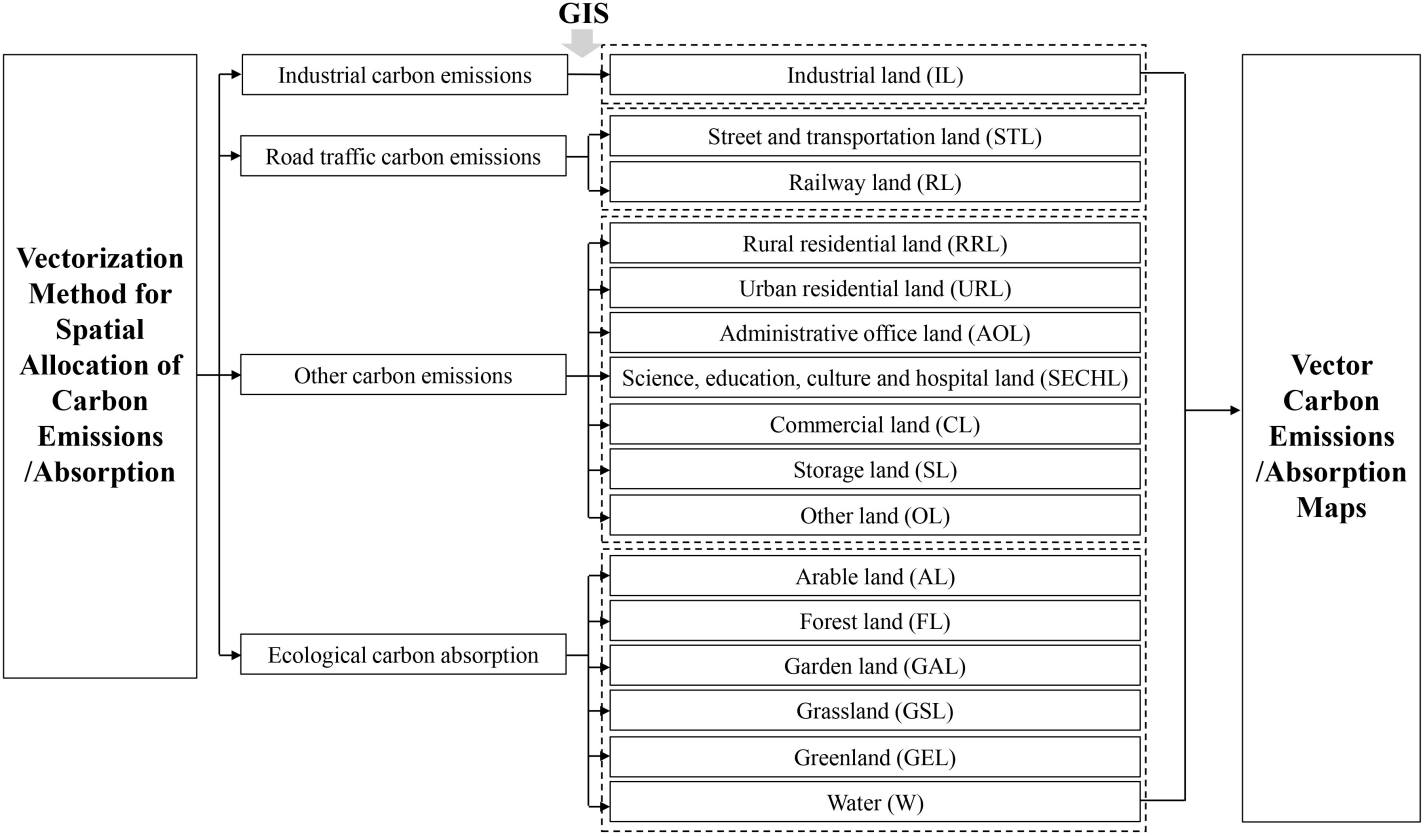
The process of mapping land patch level carbon emissions.

In the process of drawing the vector carbon emissions map, the carbon source vector database is firstly established on GIS as the basis of carbon emissions allocation. As shown in Figure 2, the database is divided into four parts: industrial, road traffic, other types of carbon emissions, and ecological carbon absorption. Then, using the vectorization method of carbon emissions spatial allocation, carbon emissions are spatially-allocated to three basic objects: industry, road, and other land patches. All data are converted to vector data and adjusted to the same coordinate. Table 4 shows the detailed allocation results based on 33,232 land patches in Zhangdian.

**Table 4:** The detailed allocation results of 33,232 land patches in Zhangdian.

**Sources of carbon emissions/absorption**	**Basic objects**	**Corresponding land use types**
Manufacturing industries	2,421 points of different industries	Industrial land (IL)
Traffic emissions	Road system	Street and transportation land (STL)
Railway emissions	Railway line	Railway land (RL)
Rural residents life electricity consumption	2,536 land patches	Rural residential land (RRL)
Urban residents life electricity consumption	1,063 land patches	Urban residential land (URL)
Public service and management organization	377 land patches	Administrative office land (AOL)
	572 land patches	Science, education, culture and hospital land (SECHL)
Commerce	2,199 land patches	Commercial land (CL)
Warehousing and postal services	770 land patches	Storage land (SL)
Other activities	1,285 land patches	Other land (OL)
Agricultural activities	4,975 land patches	Arable land (AL)
Tree activities	6,556 land patches	Forest land (FL)
Orchard activities	2,138 land patches	Garden land (GAL)
Grass activities	975 land patches	Grassland (GSL)
Green space activities	430 land patches	Greenland (GEL)
Water activities	984 land patches	Water (W)

#### Spatial autocorrelation of carbon emissions

##### Global spatial autocorrelation analysis method

Spatial autocorrelation can explore the spatial distribution law of carbon emissions, and analyze the spatial correlation degree between a certain attribute value of a land patch and its surrounding land patches in the study area, as well as the statistical distribution law of land patches and the interdependence between data. It is of great significance for spatial correlation analysis of carbon emissions at the land patch level. Therefore, its applications are more and more extensive, represented by global Moran's I (48, 49). Moran's I is mainly used to test whether there are similarities or differences between neighboring areas in the whole region (49). The value of Moran's I is distributed between [−1, 1] (50). Moran's I >0 indicates a positive correlation, which refers to the agglomeration of high value and high value or low value and low value. The closer the value is to 1, the stronger the agglomeration degree is. Moran's I < 0 indicates a negative correlation, which means that the high value is adjacent to the low value or the low value is adjacent to the high value. The closer it is to −1, the greater the difference. Moran's I is equal to 0, indicating no correlation (51, 52). The formula of Moran's I is shown in Equation (8).


(8) $$\begin{array}{l} \text{I}=\frac{n\overset{n}{\underset{i=1}{\sum }}\overset{n}{\underset{j=1}{\sum }}w_{ij}\left(x_{i}-\bar{x}\right)}{\overset{n}{\underset{i=1}{\sum }}\overset{n}{\underset{j=1}{\sum }}{w_{ij}\left(x_{i}-\bar{x}\right)}^{2}} \end{array}$$

Where n represents the total number of land patches within the study area, *w*_*ij*_ is the spatial weight, *x*_*i*_ is the variable observed in patch i, *x*_*j*_ is the variable observed in patch j, *x* is the mean of the observed value.

Moran's I is meaningful only after the significance test. Significance levels in this study were determined by *p*-value tests of standardized z-values (53, 54). The formulas of the significance test are as follows:


(9) $$\begin{array}{lll} \text{Z} & = & \frac{\text{Mora}{\text{n}}^{\text{′}}\text{s I}-\text{E}\left(\text{I}\right)}{\sqrt{VAR\left(I\right)}} \end{array}$$

(10) $$\begin{array}{lll} \text{E}\left(\text{I}\right) & = & -\frac{1}{n-1} \end{array}$$

(11) $$\begin{array}{lll} \text{VAR}\left(\text{I}\right) & = & \frac{n^{2}w_{1}+nw_{2}+3w_{0}^{2}}{w_{0}^{2}\left(n^{2}-1\right)} \end{array}$$

(12) $$\begin{array}{lll} w_{0} & = & \sum_{i=1}^{n}\sum_{j=1}^{n}w_{ij} \end{array}$$

(13) $$\begin{array}{lll} w_{1} & = & \frac{1}{2}\sum_{i=1}^{n}\sum_{j=1}^{n}{\left(w_{i}+w_{j}\right)}^{2} \end{array}$$

(14) $$\begin{array}{lll} w_{2} & = & \sum_{i=1}^{n}\sum_{j=1}^{n}{\left(w_{i}+w_{j}\right)}^{2} \end{array}$$

Where E (I) represents the expected value of Moran's I. *w*_*i*_ and *w*_*j*_ represents the sum of row i and column j in the spatial weight matrix respectively. If the Z value is >1.96, it indicates that there is a positive spatial autocorrelation at the significance level of 5%, which is manifested as high-value or low-value congeneric clustering. If the Z value is < -1.96, it indicates that there is a negative spatial correlation at the significance level of 5%, which indicates high-value and low-value heterogeneous clustering (55, 56). In other cases, it indicates that the attribute characteristics do not have correlation and are randomly distributed.

##### Local spatial autocorrelation analysis method

The global spatial autocorrelation studies the correlation within the entire spatial system. However, in the study area, local land patches may be randomly distributed even if there is a global correlation (57). The limitation of Moran's I is that if there is a positive correlation between carbon emissions in some regions and a negative correlation in the other region, the two may offset each other and reduce Moran's I. The local spatial autocorrelation analysis, also known as LISA (Local Indicators of Spatial Association), can test whether there are similar or different clusters between local areas and surrounding areas by observing the unstable characteristics of local space (58). On the one hand, the LISA deeply reflects the internal characteristics of global autocorrelation, on the other hand, it shows which regions are similar and which regions are different from each other. It identifies different spatial association patterns in different local spaces in the whole world. Specific analysis can be carried out on high-value agglomeration regions, low-value agglomeration regions, and high-low-value heterogeneous agglomeration regions (59). Studies have shown that the local space autocorrelation analysis is generally analyzed by Moran scatter plot, LISA clustering, and Getis-Ord Gi^*^ statistics (60).

**Figure 3:**
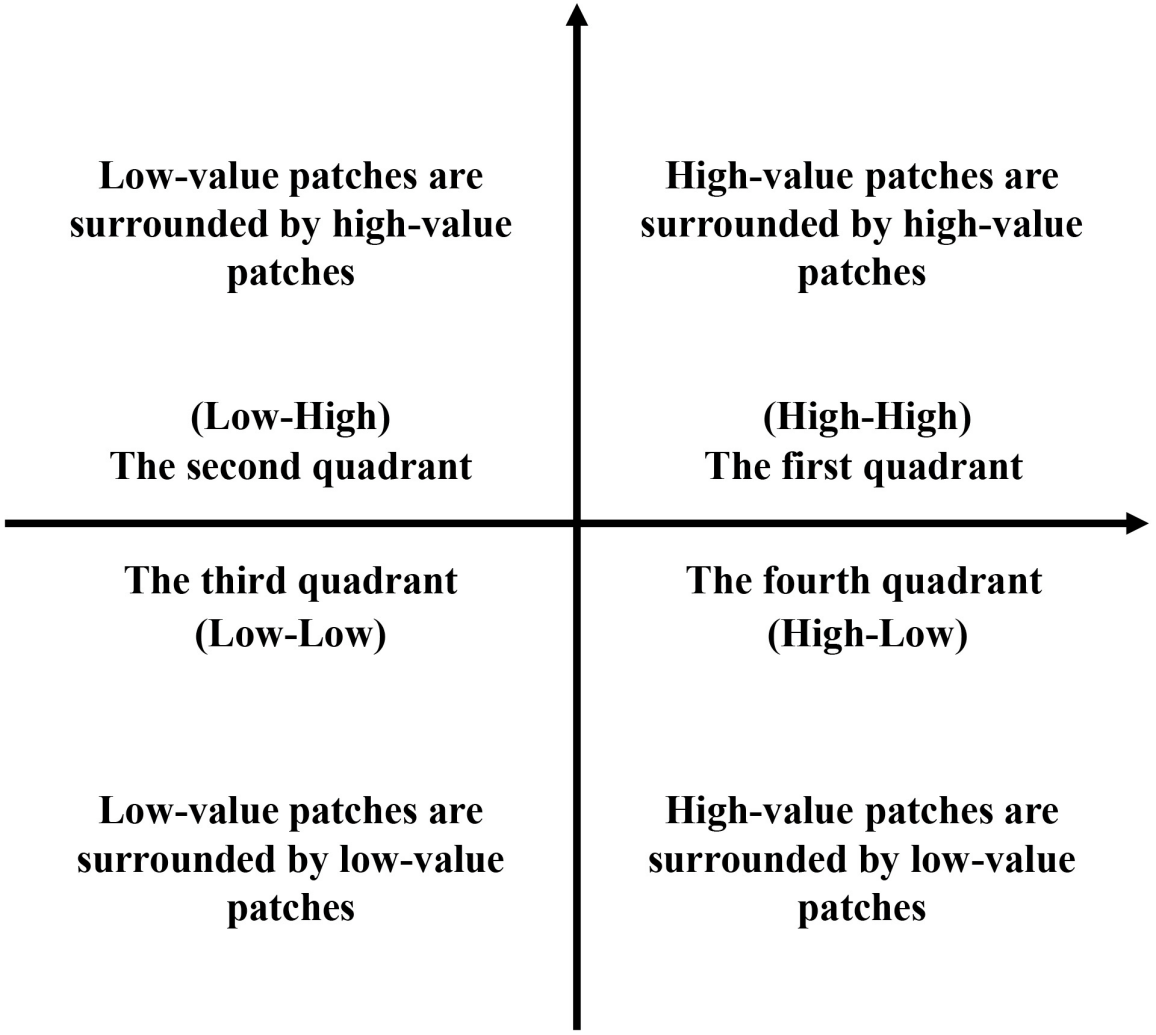
Four quadrants of the Moran scatter plot.

*Moran scatter plot*. The Moran scatter plot is a two-dimensional diagram that truly presents the spatial distribution of the research object (61). In the Cartesian rectangular coordinate system, the coordinate map is drawn with the observation vector as the horizontal axis and the weighted average of the values of the adjacent areas of the observation values as the vertical axis. The Moran scatter plot includes four quadrants, each representing four local spatial agglomeration modes between spatial units and their adjacent units. Among them, the units in the first quadrant and the third quadrant are positively correlated with their surrounding unit space, and the regions where they are located are homogeneous, while the units in the second quadrant and the fourth quadrant are negatively correlated with their surrounding unit space, and the regional heterogeneity is prominent (62) (Figure 3).

*LISA clustering*. LISA clustering can be used to measure the degree of spatial agglomeration between the attribute values of each land patch and its surrounding land patches, and it will clearly show the distribution characteristics of all land patches (63). Through the LISA clustering, the type of each land patch can be counted, and then the relationship between each land patch and the surrounding land patches can be determined.

## Results

### Vector carbon emissions and absorption map based on land patches

**Figure 4:**
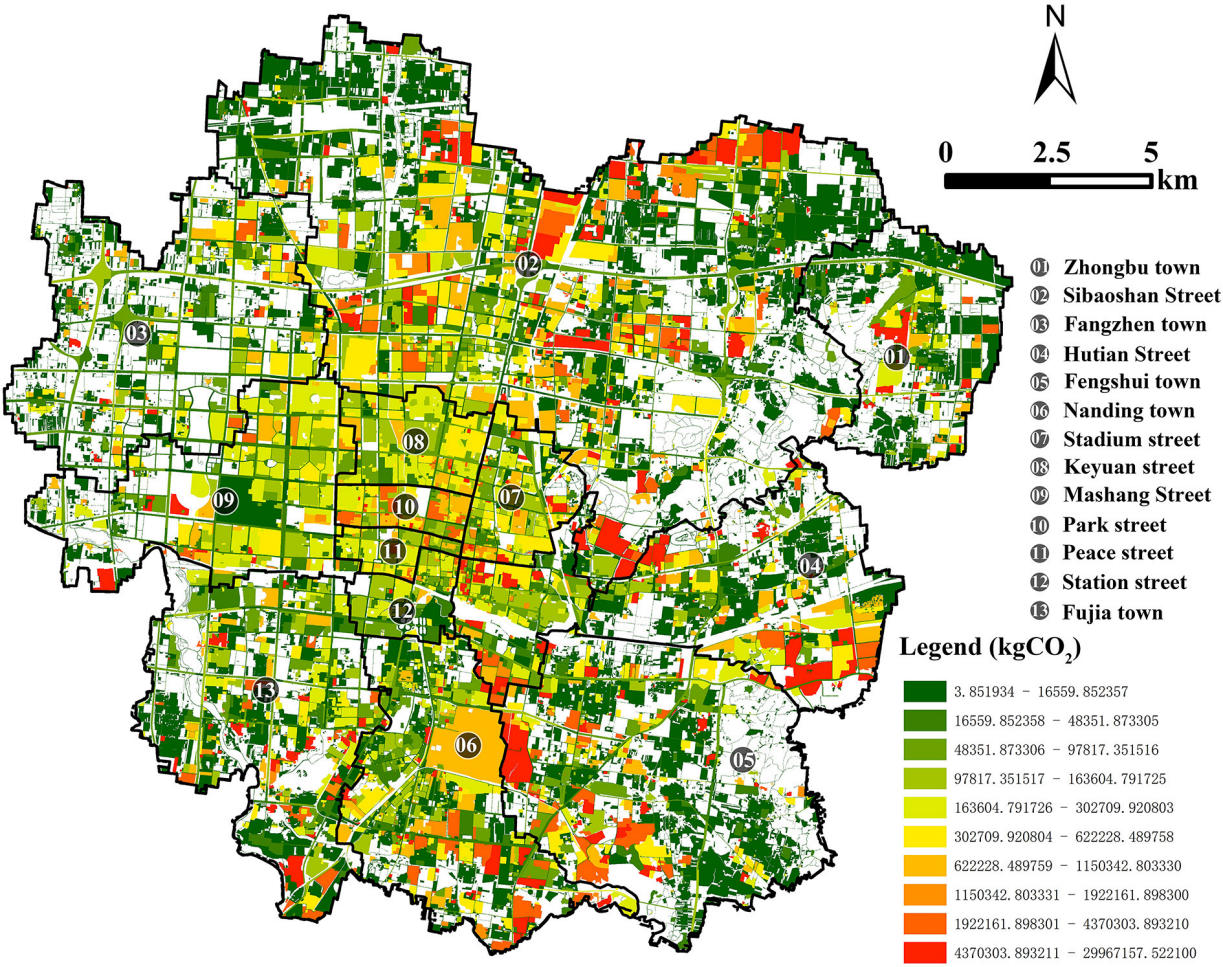
Carbon emissions map of Zhangdian.

The spatial distribution of carbon emissions from land use after allocation is displayed through the GIS framework, and a visual map is generated. The total annual carbon emissions of each land patch in Zhangdian are shown in Figure 4, and the carbon emissions are distributed between 3.85 and 3.00 × 10^7^ kg, showing a large range of changes. In 2021, the total carbon emissions of Zhangdian are 4.76 × 10^9^ kg, of which the area of industrial land only accounts for 13.8% of the land area, but the carbon emissions intensity is much higher than that of other types of land, accounting for 70.16% of the overall carbon emissions, which is the main source of carbon emissions. Additionally, the urban residential land carbon emissions account for 7.67%, commercial service facilities land carbon emissions account for 4.98%, other land carbon emissions account for 4.85%, science, education, culture and health land carbon emissions account for 4.48%, public service and management organization land carbon emissions account for 3.41%, and other emissions sources account for a relatively low proportion.

The spatial distribution of carbon emissions shows an obvious imbalance. As can be seen from the map of the red area, areas of high carbon emissions are mainly concentrated in the east and north of the study area, including Sibaoshan, Hutian, and Fengshui. On the one hand, the industrial land in these three regions is relatively concentrated. On the other hand, the carbon emissions of industrial energy consumption are much higher than that of other land use types and even much higher than that of core urban areas where population and human activities are most concentrated, which is the main source of carbon emissions. From the green area of the map, it can be seen that the low-carbon emissions areas are mainly distributed in Zhongbu, Fujia, and Fangzhen on the periphery of the study area, which has relatively more rural residential land (Figure 4).

**Figure 5:**
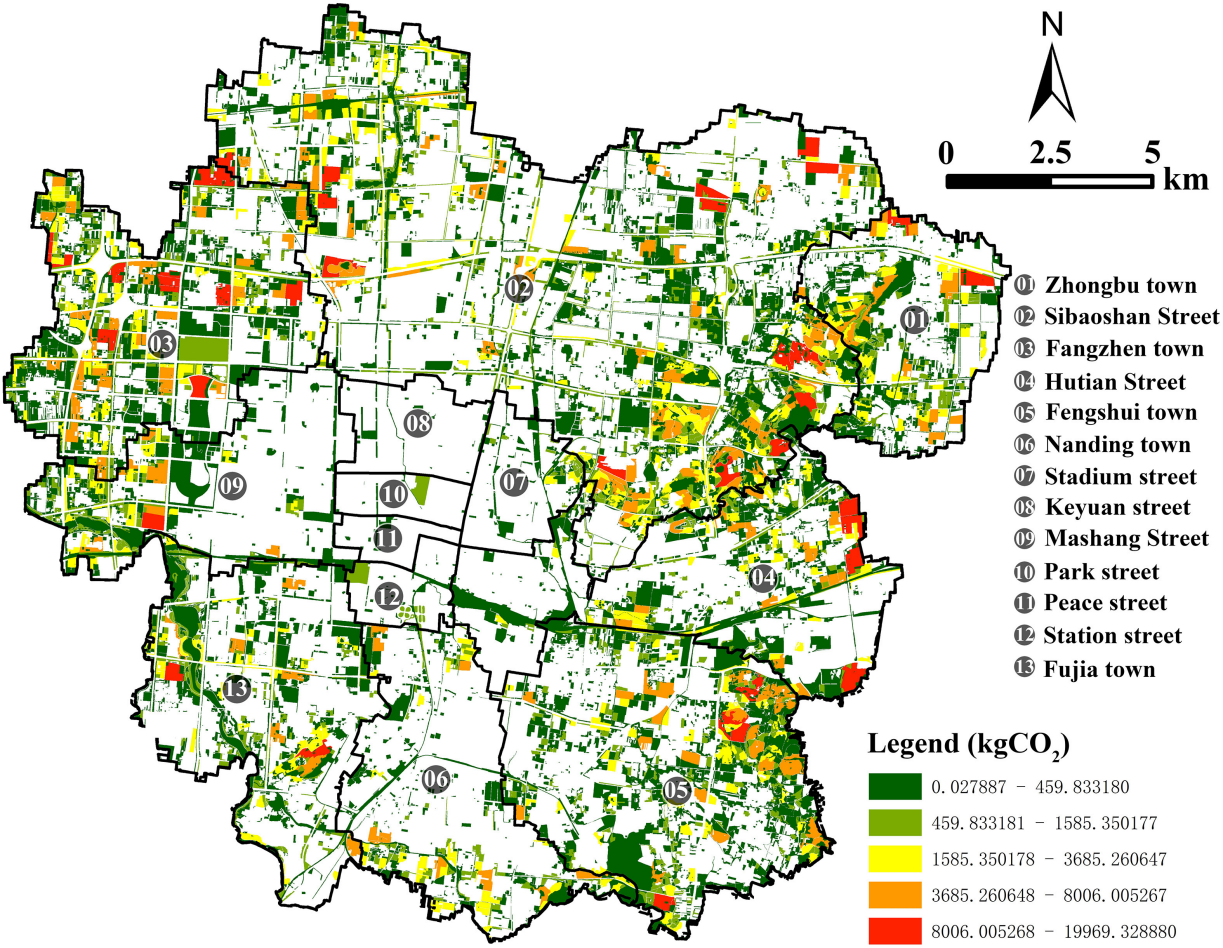
Carbon absorption map of Zhangdian.

From the perspective of carbon absorption, the spatial distribution of carbon absorption from land use after allocation is displayed through the GIS framework, and a visual map is generated. The total annual carbon absorption of each patch in Zhangdian is shown in Figure 5, and the carbon absorption is distributed between 0.03 and 2.00 × 10^4^ kg, with a relatively small variation range. The total carbon absorptions of Zhangdian are 4.28 × 10^6^ kg in 2021, of which the carbon absorption of forest land accounts for 98.56%, which is the main land use type of carbon absorption in Zhangdian. Other land use types have smaller carbon uptake.

The spatial distribution of carbon absorption also showed an obvious imbalance. As can be seen from the map of the red area, the high carbon absorption areas are mainly concentrated in Sibaoshan street, Zhongbu, Hutian, and Fengshui in the east of the study area, as well as Fangzhen, Mashang, and Fujia in the West. On the one hand, the area of the seven streets is large. On the other hand, the seven streets are mainly located at the edge of the core urban area, with large areas of forest land, garden land, and other land, and the overall carbon absorption level is relatively high. As can be seen from the green area of the map, low-carbon absorption areas are mainly distributed near the core urban areas where population and human activities are most concentrated, as well as the parks, road greening, and water system within the core urban areas, including Keyuan, Park, Peace, Station, and Stadium (Figure 5).

### Spatial autocorrelation of carbon emissions

#### Global spatial autocorrelation

When analyzing the global spatial autocorrelation, both the carbon emission land use type and the carbon absorption land use type are regarded as the carbon emission land use type, and the carbon emission value of the carbon absorption land use type is regarded as a negative number to ensure the uniformity and reliability of the results. As can be seen from Table 5, the results showed that the land patch level global Moran' I was 0.138, with the Mean was 0.0000. The *P* < 0.01 indicated that they had passed the significance test at the 1% level, and the Z value >1.96 indicated that they had passed the Z test. The results showed that carbon emissions in Zhangdian had a significant positive correlation, which conformed to the first law of geography. In other words, with the increasing distance between two land patches, the degree of correlation between them gradually decreases, while with the further increase of the distance between two land patches, they no longer correlate with each other, showing the characteristics of random distribution.

**Table 5:** The global Moran' I of land patch level carbon emissions.

**Moran' I**	**Mean**	**Standard error**	**z-value**	***P*-value**
0.138	0.0000	0.00146	76.92	0.002

#### Local spatial autocorrelation

The global spatial autocorrelation can only analyze the correlation of carbon emissions on the whole. In this study, Moran scatter plot and LISA clustering were calculated by GeoDa software to further analyze the distribution rules of carbon emissions from land use in different regions in Zhangdian.

As can be seen from Table 6, excluding Not Significant and Neighborless types, the distribution of land use carbon emissions in Zhangdian is not evenly distributed in the four quadrants. Among them, the number of “High-High” land patches is 2,342, accounting for 7.05% of all land patches in Zhangdian. The number of “Low-High” land patches was 1,184, accounting for 3.56%. The number of “Low-Low” land patches was 5,692, accounting for 17.13%. The number of “High-Low” land patches was 2,190, accounting for 6.59%.

**Table 6:** Quadrants distribution statistics of land patch carbon emissions in Zhangdian.

**High-high**		**Low-high**		**Low-low**		**High-low**		**Not significant**		**Neighborless**	
**Number**	**Ratio**	**Number**	**Ratio**	**Number**	**Ratio**	**Number**	**Ratio**	**Number**	**Ratio**	**Number**	**Ratio**
2,342	7.05%	1,184	3.56%	5,692	17.13%	2,190	6.59%	21,820	65.66%	4	0.01%

**Figure 6:**
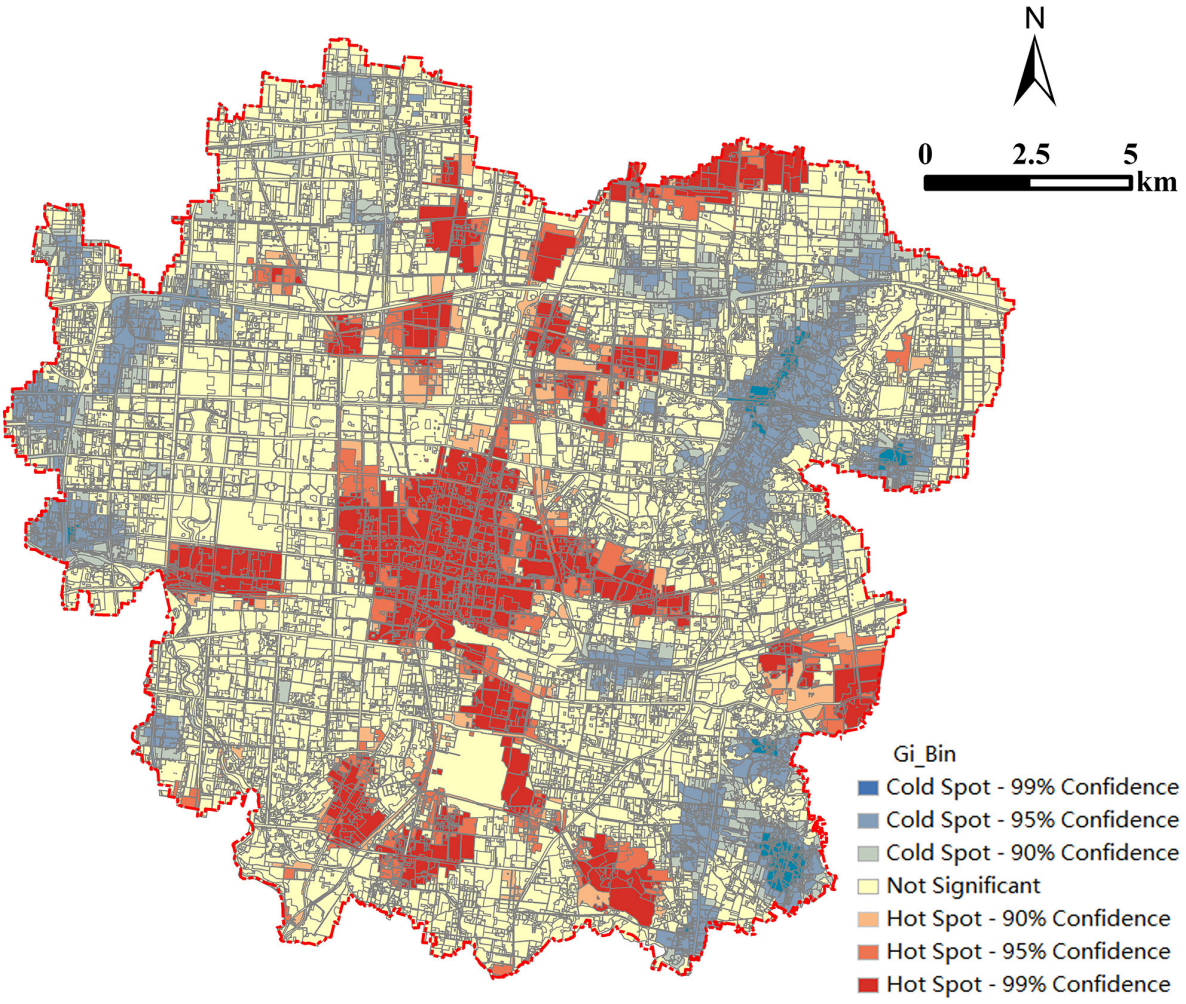
The Getis-Ord Gi* statistics of land patch level carbon emissions.

The Getis-Ord Gi^*^ statistics is consistent with the principle of LISA clustering, but the scope is more accurate. This tool is used to identify spatial clusters of statistically significant high values (hot spots) and low values (cold spots). In effect, it indicates whether the observed spatial clustering of high or low values is more pronounced than we would expect from a random distribution of these same values. It can be seen from Figure 6 that the red areas are “High-High” agglomeration areas, mainly the core urban area with the most concentrated population and activities and the surrounding industrial land, including Sibaoshan, Keyuan, Stadium, Peace, Park, Station, Nanding, Mashang, and Hutian. The area with the concentration of forest land and grassland showed the characteristics of “Low-Low” agglomeration, including Fangzhen, Zhongbu, Fengshui, and Sibaoshan. The orange-red areas are the “High-Low” agglomeration areas, mainly around the “High-High” agglomeration areas, which are consistent with the spatial distribution of the “High-High” agglomeration areas. The light-blue areas are the “Low-High” agglomeration areas, mainly around the “Low-Low” agglomeration areas, which are consistent with the spatial distribution of the “Low-Low” agglomeration areas. These two types showed the characteristics of “High-Low” or “Low-High” agglomeration due to the lack of dominant function of regional land use. This indicates that there is a spatial diffusion or radiation effect between the “High-Low” agglomeration area of land patches carbon emissions and the surrounding land patches, which makes the spatial difference between the two areas smaller and smaller. Additionally, there are certain similarities between adjacent patches. From the distribution and changes of colors shown in Figure 6, it can be seen that the carbon emissions of neighboring land patches are mostly distributed in the same range, showing a certain regional agglomeration.

### Comparison with other method

Taking Stadium in Zhangdian as an example, the mapping results of this study are compared with another research method (27). All maps are based on the same total energy consumption data from the 2021 Zibo statistical yearbook. The detailed differences between the two methods are shown in Table 7. This comparison shows that the estimation results obtained by this study are more accurate at the land patch level and can better support urban planning at the medium and micro scale.

**Table 7:** Detailed differences of the two carbon emissions mapping methods.

**Method**	**Allocation weight**	**Carbon emissions from roads**	**Carbon absorption**	**Basic unit**	**Land use type**	**Source**
(a)	Energy consumption data, population, building area, industry categories, etc.	No	No	300 × 300m	4	(29)
(b)	Energy consumption data, land area, population, building area, road area, road length, traffic flow, industry categories, etc.	Yes	Yes	Land patches	16	This study

**Figure 7:**
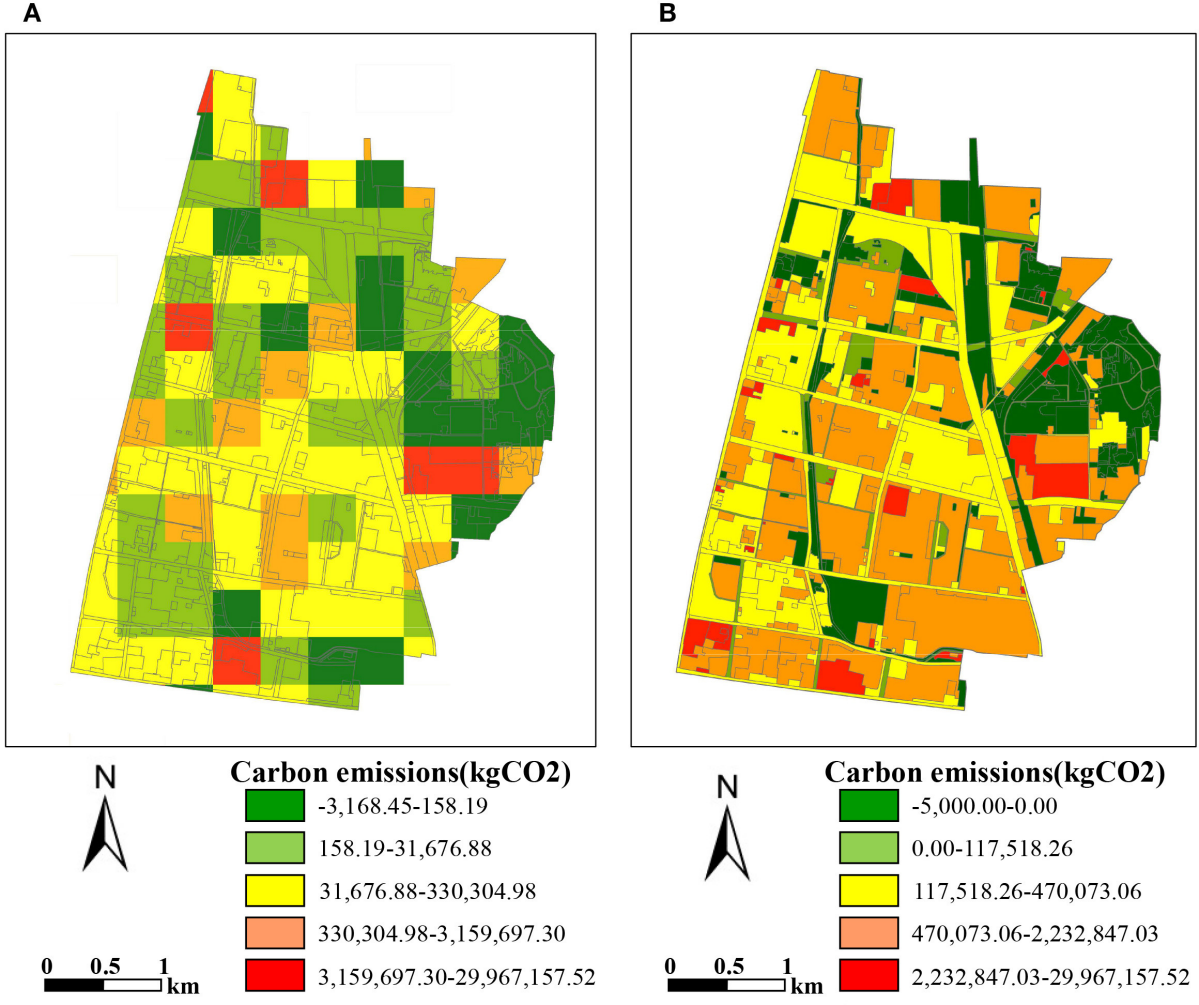
Comparison of the carbon emissions mapping results with other method. **(A)** Gridded carbon emissions map with 300 × 300m. **(B)** Vector carbon emissions map of this study.

The allocation results of each method are shown in Figure 7. Overall, both maps show a similar spatial distribution of high carbon emissions in the north and southeast and low carbon emissions in the middle and east to some extent. This may have something to do with a large amount of industrial land in the southeast and north. However, compared with the grid map shown in Figure 7A, the vector carbon emissions map in this study provides carbon emissions of land patches with clear geographical locations and boundaries, which can provide more intuitive support for urban planning. The main difference between Figures 7A,B lies in the clear location and scale of high-carbon emissions land patches in the southeast region, which happens to be the industrial land in the region. Through actual investigation, it is found that these land patches have higher energy consumption and carbon emissions, which is more consistent with the results of our method. This comparison shows that the estimation results obtained by this method are more accurate at the land patch level. In conclusion, different from previous allocation methods, the method proposed in this study considers the influencing factors of different types of carbon emissions which can make the mapping result more consistent with the actual situation. It can better identify the difference in carbon emissions of specific land use types and estimate the carbon emissions of each land patch more accurately.

## Discussion

### Applications

**Figure 8:**
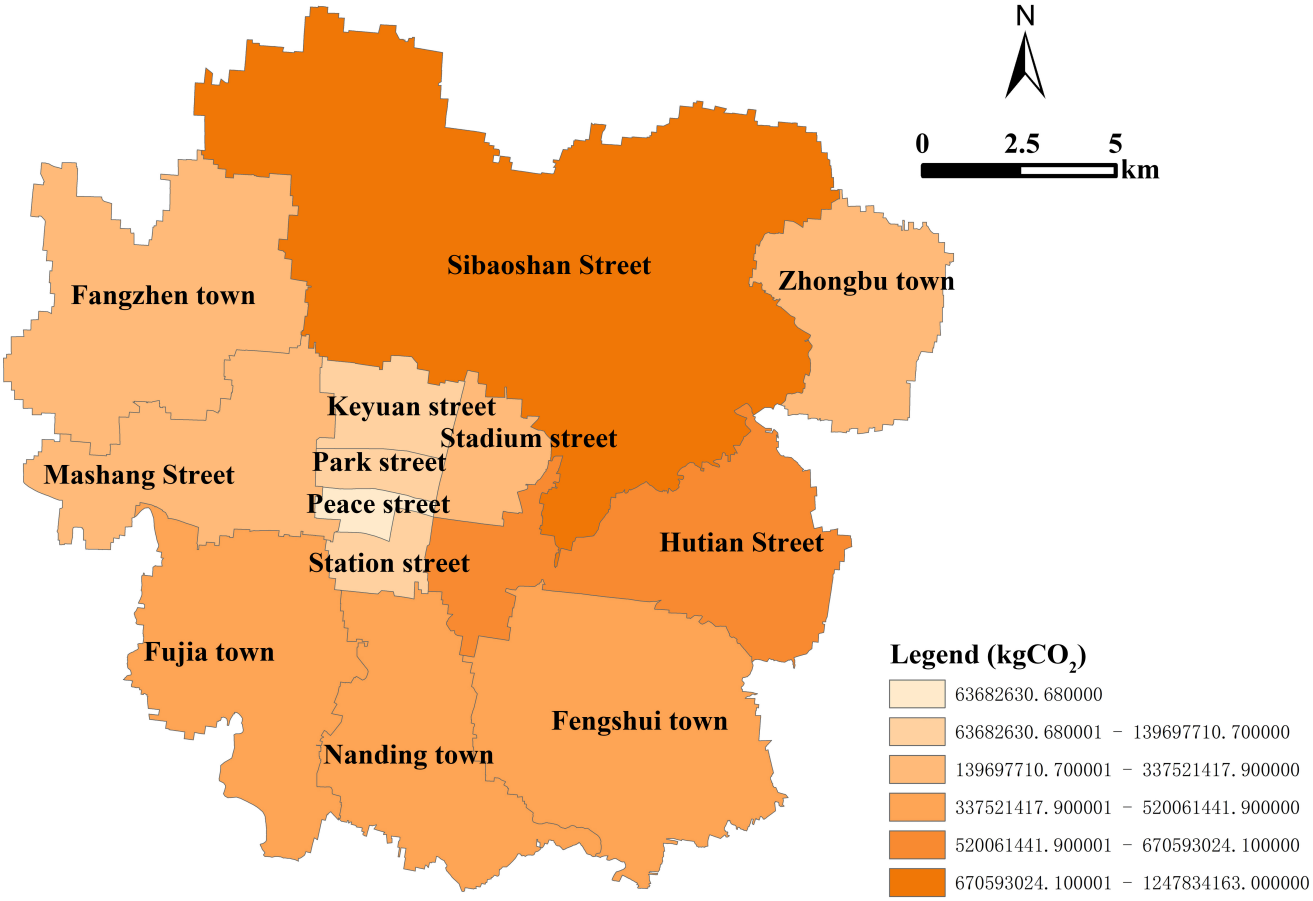
Carbon emissions of subdistricts in Zhangdian.

In recent years, how to accurately allocate and visualize the overall carbon emissions of each land patch has always been a concern (Yang et al., 2021), which plays an important role in guiding urban carbon emissions reduction policies and low-carbon planning (64). Taking Zhangdian as an example, the specific application is reflected in the following two aspects. On the one hand, the carbon emissions of administrative units such as streets, towns, and villages can be calculated without losing accuracy. By determining the key carbon emissions administrative regions, the carbon emissions reduction targets are divided into basic administrative units. Figure 8 shows the total carbon emissions of 13 streets in Zhangdian. Sibaoshan has the highest carbon emission in 2021, with carbon emissions of 1.25 × 10^9^ kg, which is significantly higher than that of other streets, followed by Hutian with a carbon emission of 6.71 × 10^8^ kg. Peace has the lowest carbon emissions, with carbon emissions of 6.37 × 10^7^ kg. The carbon emissions from high to low is Sibaoshan, Hutian, Fengshui, Nanding, Fujia, Fangzhen, Stadium, Mashang, Zhongbu, Park, Station, Keyuan, and Peace. Additionally, it is found that the two towns with high carbon emissions are also important industrial towns in Zhangdian. These towns are the focus of Zhangdian to reduce carbon emissions by adjusting industrial structure or improving energy efficiency. Depending on the proportion of carbon emissions, they should be responsible for about 60% of the overall carbon emissions reduction target of Zhangdian.

**Figure 9:**
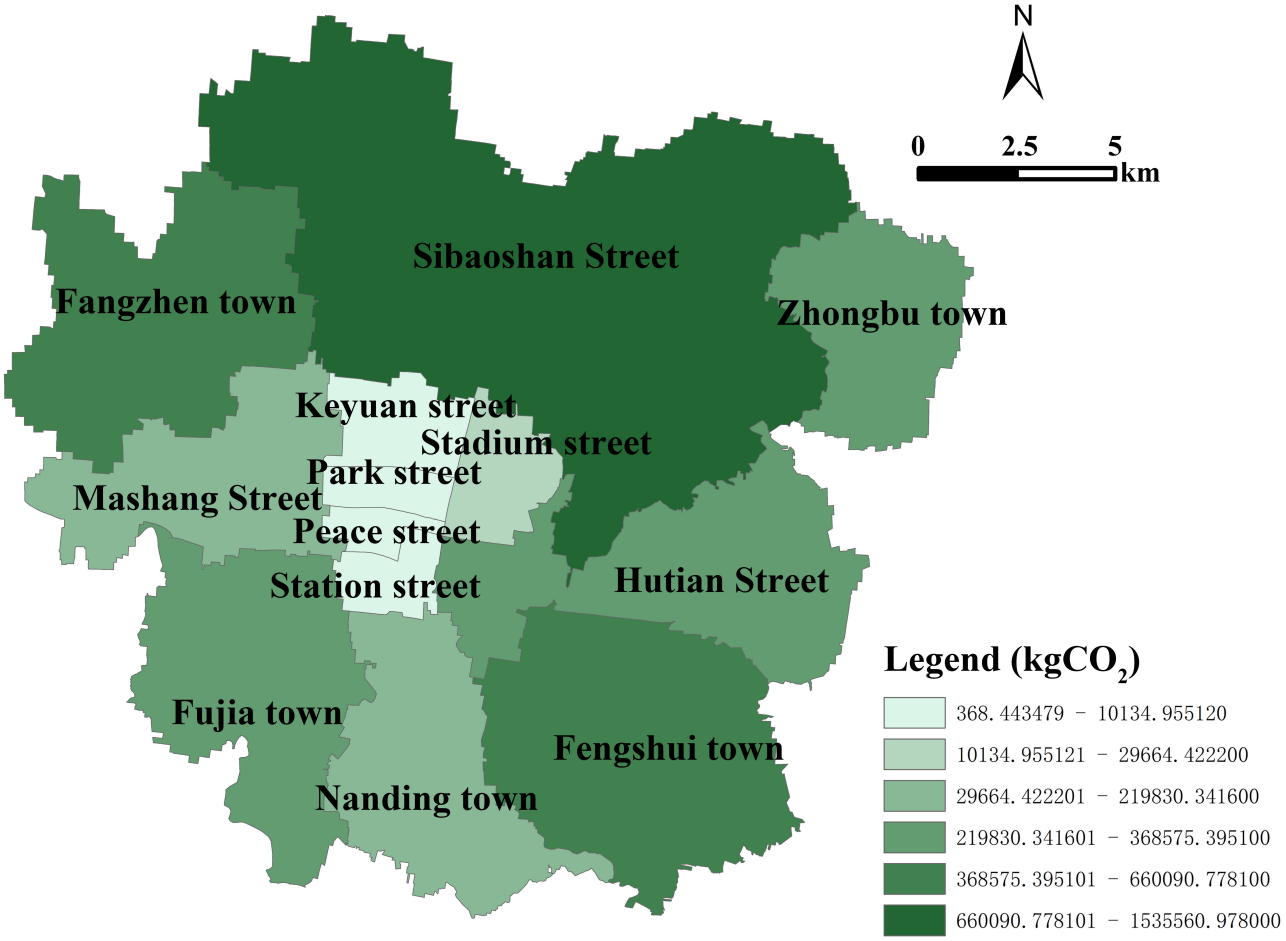
Carbon absorption of subdistricts in Zhangdian.

Figure 9 shows the total carbon absorption of 13 streets in Zhangdian. In 2021, Sibaoshan had the highest carbon absorption, with 1.54 × 10^6^ kg. The second is Fangzhen, with a carbon absorption of 6.60 × 10^5^ kg. Peace has the smallest carbon absorption, with a carbon absorption of 3.68 × 10^2^ kg. The carbon absorption from high to low is Sibaoshan, Fangzhen, Fengshui, Hutian, Zhongbu, Fujia, Nanding, Mashang, Stadium, Station, Keyuan, Park, and Peace. Additionally, it was found that the two towns with higher carbon absorption were also important forest land reserves in Zhangdian. However, compared with Sibaoshan, Fangzhen has a lower vegetation coverage rate, the highest net emissions, and the highest environmental pressure. Therefore, specific measures must be seriously considered and implemented.

**Figure 10:**
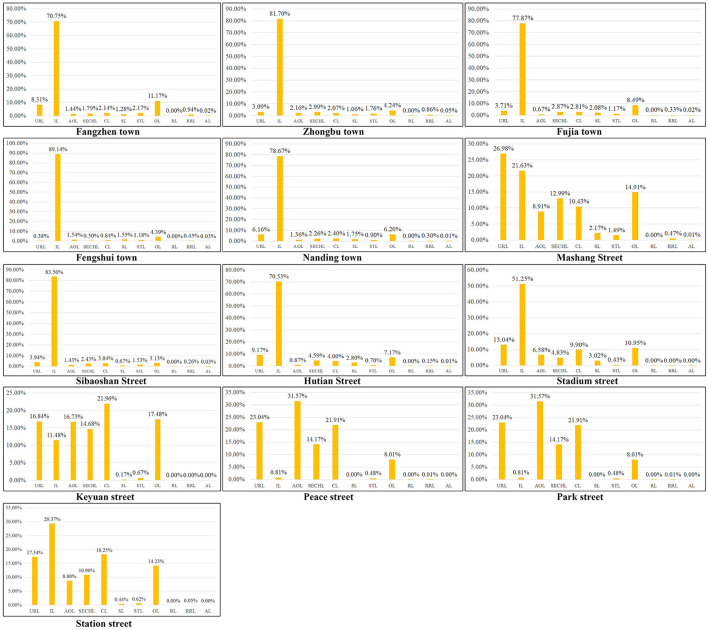
Carbon emissions ratio of land patch in different streets of Zhangdian.

On the other hand, the characteristics of carbon emissions of different land use types in different streets can be compared, and further targeted urban planning suggestions can be put forward. According to Figure 10, Fengshui has the highest proportion of industrial carbon emissions, accounting for 89.14%, far higher than the average level of 70.16% in Zhangdian. The top five streets with the highest proportion of industrial carbon emissions include Sibaoshan, Hutian, Fengshui, Nanding, and Fujia, account for 83.56, 70.53, 89.14, 78.67, and 77.87% respectively, both higher than the average level of Zhangdian. The streets with low carbon emissions, such as Stadium, Station, Keyuan, Park, and Peace, accounting for relatively low industrial carbon emissions, which are 51.25, 29.37, 11.48, 16.21, and 0.81% respectively, lower than the average level of Zhangdian. These streets are in the core urban areas with the most concentrated population and activities, and the proportion of buildings carbon emissions such as residential buildings and commercial buildings are relatively high. Specifically, among the carbon emissions of different land use types in the 13 streets of Zhangdian, Mashang has the highest proportion of urban residential land (URL) carbon emissions, accounting for 26.98%, and Fengshui has the highest proportion of industrial land (IL) carbon emissions, accounting for 89.14%, Peace has the highest proportion of administrative office land (AOL) carbon emissions, accounting for 31.57%, Park has the highest proportion of science, education, culture and hospital land (SECHL) carbon emissions, accounting for 23.61%, Keyuan has the highest proportion of commercial land (CL) carbon emissions, accounting for 21.96%, Stadium has the highest proportion of storage land (SL) carbon emissions, accounting for 3.02%, and Fangzhen has the highest proportion of street and transportation land (STL) carbon emissions and Other land (OL) carbon emissions, The proportion is 2.17 and 11.02% respectively. Railway land (RL) carbon emissions in Keyuan is the highest, accounting for 16.67%. The proportion of other land types is relatively low, < 1%.

**Figure 11:**
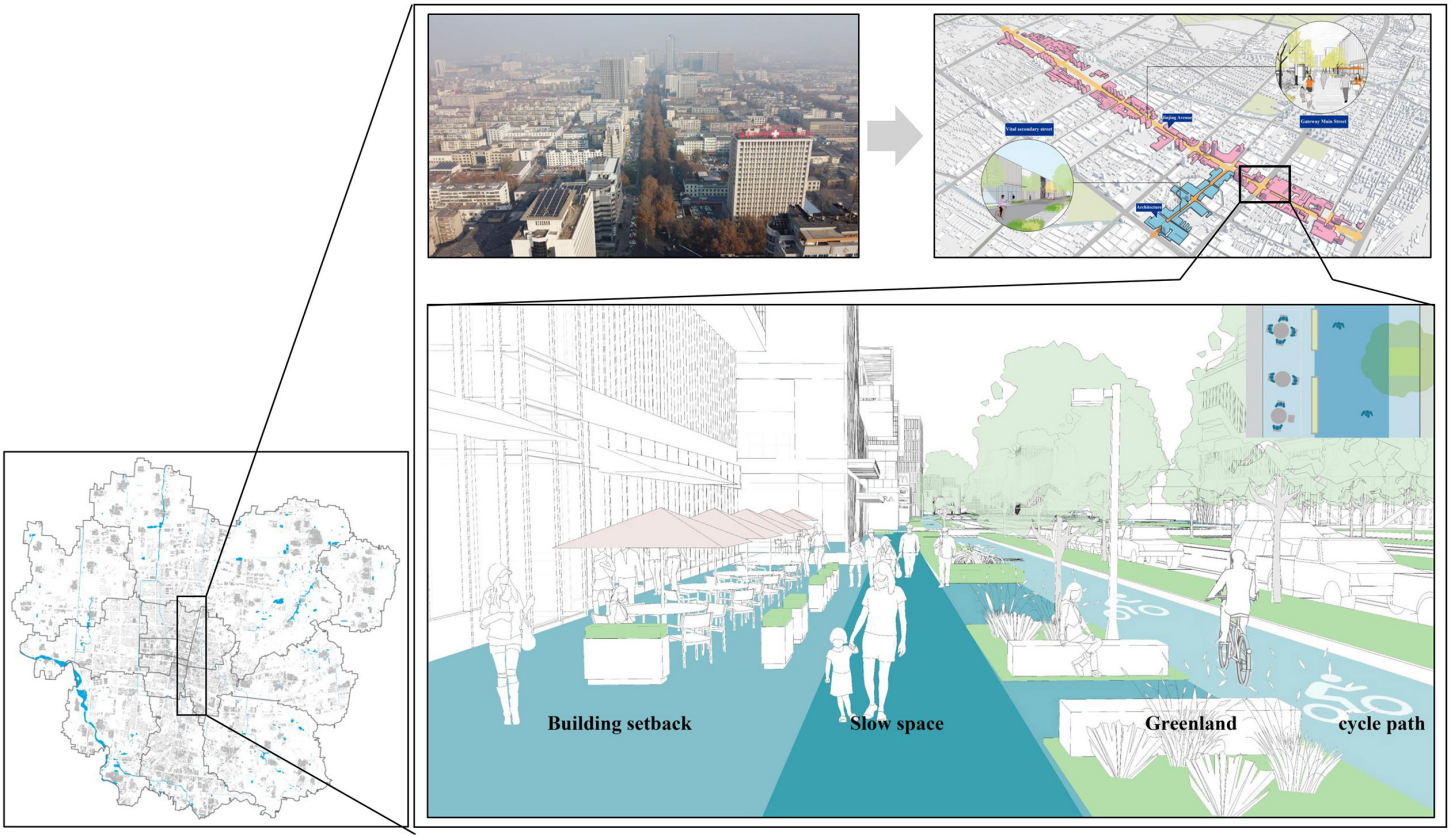
Low-carbon and health-oriented Jinjing Avenue design scheme.

Specific planning suggestions include: first, increasing vegetation coverage is not only the need to reduce carbon emissions but also the need to improve the living environment. Studies have confirmed that the concentration of pollutants such as carbon emissions has a strong positive correlation with the morbidity and mortality of respiratory diseases, cardiovascular and cerebrovascular diseases (65). Therefore, it is suggested that in the urban centers, such as Stadium Street, Peace Street, Park Street, Station Street, and Keyuan Street, the huge contradiction between the demand for artificial land and vegetation land should be fully considered, and carbon emissions can be absorbed and the pollutant concentration reduced by increasing the roof greening, while in the suburbs, such as Hutian Street, Nanding Town, and Sibaoshan Street, carbon absorption can be improved by increasing the area of vegetation (66, 67). Second, effective physical activity is one of the important ways to prevent and reduce chronic diseases. Walking can not only reduce traffic carbon emissions but also reduce the incidence of chronic diseases and dependence on drug control. Low-carbon and health-oriented urban planning should consider physical environments that are suitable for walking, cycling, and physical exercise, and ensure that physical exercise, walking and cycling are easily accessible to all households by encouraging people to spend more time outdoors by adding more comfortable pedestrian-level open Spaces (68, 69). Taking the planning of Jinjing Avenue in Heping Street as an example, the low-carbon and health-oriented urban planning redesigns the backward space of the building, integrates green space, slow walking space and bicycle path, and forms a high-quality traffic space with pleasant scale, which promotes more physical activity, so as to prevent and reduce diseases (Figure 11).

Third, the concentration of pollutants such as carbon emissions in cities has important effects on respiratory diseases. Low-carbon and health-oriented urban planning can affect the concentration and spatial distribution of pollutants and have certain effects on respiratory health results by adjusting the built environment factors, such as optimizing the layout of industrial land and residential land, controlling the building form, developing ventilation paths, etc. (70, 71). In Zhangdian, low-carbon and health-oriented urban planning should make full use of green space, follow the dominant wind direction, and create a multi-level ventilation corridor system. In the ventilation corridor area, green Spaces such as water system, park and woodland should be strictly protected, and the construction scale should be strictly controlled, especially the construction increment of the main air inlet, and the control of building height, building spacing and density should be strengthened. In addition, the design should also focus on areas with low wind speed, strong heat island and pollution prone agglomeration to further reduce urban carbon emissions. Fourth, industries need to be removed from areas with high population density (72). Peace, Stadium, Station, and Park are the core urban areas of Zhangdian. Since the proportion of industrial land is much lower than in Sibaoshan, carbon emissions are significantly lower and the pressure of carbon emissions reduction is also relatively lower. Hutian and Sibaoshan have similar proportions of industrial land, while Sibaoshan has higher vegetation coverage and lower population density. Therefore, differentiated carbon reduction strategies need to be proposed. Overall, the adjustment of industrial structure is undoubtedly the most critical factor to reduce carbon emissions, improve the living environment, and promote the public's physical and mental health (73, 74). As for the other streets, although most industrial lands are distributed in these zones, they are far from the core urban areas and have a higher coverage of vegetation, resulting in relatively low environmental pressures compared to other areas (75). Therefore, each street should take differentiated measures according to the characteristics of the region and the main problems faced.

Furthermore, the vector carbon emissions map can provide the carbon emissions of each land patch and the impact factors of carbon emissions. This is important for low-carbon and health-oriented urban planning because the map can visualize how and why the function, form, density, and other indicators of existing land patches affect carbon emissions and public health. Studies have proved that land use type, building density, and floor area ratio have important effects on carbon emissions and public health (76–79). However, the quantitative relationship between them and the underlying mechanism remains unclear (80). The vector carbon emissions map provides sufficient data to further study the relationship between carbon emissions and land use characteristics (64). These data can also provide additional support for urban planning to achieve carbon reduction through land patch control.

### Limitations and further improvements

Uncertainties and shortcomings also exist. Firstly, there may be a certain position deviation when the industrial POI point or road system corresponds to the relevant land patches. Secondly, when allocating carbon emissions from residential and commercial sectors, this study assumes that all floors of the same type of land use are in use, and the energy consumption level of the same type of building is the same, which may be in some contrast with reality. Thirdly, the carbon emissions of Zhangdian are calculated according to the proportion of energy consumption in Zhangdian in the 2021 Zibo statistical yearbook, which may lead to some deviations. Finally, the calculation of carbon emissions is mainly based on the 2021 Zibo statistical yearbook, which only includes carbon emissions from energy consumption. According to the IPCC, carbon sources also include emissions from industrial processes, waste disposal, land-use change, and forestry change. Therefore, the total carbon emissions considered in this study may be slightly lower than the actual value.

Overall, our study significantly improves spatial resolution compared to previous studies. However, accuracy still needs to be improved, which will involve large-scale field investigations in the future. Future research should also update the basic big data related to the spatial distribution of carbon emissions, and gradually build the time series of emissions maps to provide greater support for the research on the spatial distribution of carbon emissions and policy implementation. At the same time, all land use types of the whole region need to be considered, with special attention to the detailed land use types of urban areas containing major human activities, which requires more accurate parameters and allocation algorithms.

## Conclusions

In this study, the vector maps and spatial autocorrelation of carbon emissions at the land patch level in Zhangdian, China were explored using multi-source data. The conclusions are as follows.

1. The vector carbon emissions map drawn by this method can accurately identify key emissions areas. In 2021, the total carbon emissions of Zhangdian are 4.76 × 10^9^ kg, among which industrial land carbon emissions accounted for 70.16%, mainly distributed in the east and north of the study area, including Sibaoshan, Hutian, and Fengshui. In 2021, the total carbon absorption of Zhangdian is 4.28 × 10^6^ kg, among which forest land carbon absorption accounted for 98.56%, mainly distributed in Sibaoshan street, Zhongbu, Hutian, and Fengshui in the east of the study area, as well as Fangzhen, Mashang, and Fujia in the West.
2. The results show that the Moran' I of carbon emissions at the land patch level is 0.138, which is a significant positive correlation. Among them, the number of “High-High” land patches is 2342, accounting for 7.05% of all land patches in Zhangdian. The number of “Low-High” land patches was 1,184, accounting for 3.56%. The number of “Low-Low” land patches was 5,692, accounting for 17.13%. The number of “High-Low” land patches was 2190, accounting for 6.59%.
3. Sibaoshan has the highest carbon emissions in 2021, with carbon emissions of 1.25 × 10^9^ kg, which is significantly higher than that of other streets, followed by Hutian with carbon emissions of 6.71 × 10^8^ kg. Peace has the lowest carbon emissions, with carbon emissions of 6.37 × 10^7^kg. The carbon emissions from high to low is Sibaoshan, Hutian, Fengshui, Nanding, Fujia, Fangzhen, Stadium, Mashang, Zhongbu, Park, Station, Keyuan, and Peace. In 2021, Sibaoshan had the highest carbon absorption, with 1.54 × 10^6^kg. The second is Fangzhen, with a carbon absorption of 6.6 × 10^5^kg. The Peace has the smallest carbon absorption, with a carbon absorption of 3.68 × 10^2^ kg. The carbon absorption from high to low is Sibaoshan, Fangzhen, Fengshui, Hutian, Zhongbu, Fujia, Nanding, Mashang, Stadium, Station, Keyuan, Park, and Peace.
4. This method can better identify the differences of high carbon emission land use types in different regions. URL carbon emissions of Mashang is the highest, accounting for 26.98%. IL carbon emissions in Fengshui is the highest, accounting for 89.14%; AOL of Peace is the highest, accounting for 31.57%. SECHL carbon emissions of Park is the highest, accounting for 23.61%. CL carbon emissions of Keyuan is the highest, accounting for 21.96%. SL carbon emissions of Stadium is the highest, accounting for 3.02%. STL carbon emissions and OL carbon emissions in Fangzhen are the highest, accounting for 2.17 and 11.02%, respectively. RL carbon emissions in Keyuan are the highest, accounting for 16.67%. In other land use types, carbon emissions are relatively low, accounting for < 1%.

The results of this study can provide valuable guidance for emissions reduction policies and low-carbon healthy urban planning. For city managers, the carbon emissions of each administrative unit can be calculated according to the map to identify key areas for carbon reduction. For urban planners, it can provide additional support for urban planning to achieve carbon reduction and public health.

## Data availability statement

The raw data supporting the conclusions of this article will be made available by the authors, without undue reservation.

## Author contributions

XZ wrote the main manuscript text, directed, and revised the manuscript. QL contributed to all aspects of this work. HZ, PL, and XZ conducted the experiment and analyzed the data. All authors reviewed the manuscript. All authors contributed to the article and approved the submitted version.

## Funding

This research was funded by the National Natural Science Foundation of China, Grant No. 51878393; the Youth Fund of Shandong Natural Science Foundation, Grant No. ZR2022QE151.

## Conflict of interest

Author PL was employed by Zibo Urban Planning Design Institute Co., Ltd. The remaining authors declare that the research was conducted in the absence of any commercial or financial relationships that could be construed as a potential conflict of interest.

## Publisher's note

All claims expressed in this article are solely those of the authors and do not necessarily represent those of their affiliated organizations, or those of the publisher, the editors and the reviewers. Any product that may be evaluated in this article, or claim that may be made by its manufacturer, is not guaranteed or endorsed by the publisher.
